# Environmental impacts on the diversity of methane-cycling microbes and their resultant function

**DOI:** 10.3389/fmicb.2013.00225

**Published:** 2013-08-14

**Authors:** Emma L. Aronson, Steven D. Allison, Brent R. Helliker

**Affiliations:** ^1^Department of Plant Pathology and Microbiology, University of CaliforniaRiverside, CA, USA; ^2^Department of Ecology and Evolutionary Biology, University of CaliforniaIrvine, CA, USA; ^3^Department of Earth System Science, University of CaliforniaIrvine, CA, USA; ^4^Department of Biology, University of PennsylvaniaPhiladelphia, PA, USA

**Keywords:** methane, CH_4_, methanotroph, biogeochemistry, soil, MOB, review

## Abstract

Methane is an important anthropogenic greenhouse gas that is produced and consumed in soils by microorganisms responding to micro-environmental conditions. Current estimates show that soil consumption accounts for 5–15% of methane removed from the atmosphere on an annual basis. Recent variability in atmospheric methane concentrations has called into question the reliability of estimates of methane consumption and calls for novel approaches in order to predict future atmospheric methane trends. This review synthesizes the environmental and climatic factors influencing the consumption of methane from the atmosphere by non-wetland, terrestrial soil microorganisms. In particular, we focus on published efforts to connect community composition and diversity of methane-cycling microbial communities to observed rates of methane flux. We find abundant evidence for direct connections between shifts in the methane-cycling microbial community, due to climate and environmental changes, and observed methane flux levels. These responses vary by ecosystem and associated vegetation type. This information will be useful in process-based models of ecosystem methane flux responses to shifts in environmental and climatic parameters.

## Introduction

Microorganisms have the potential to impact large-scale ecosystem functions that are relevant to the atmospheric composition of the Earth. In particular, microbial communities responsible for “narrow” processes, those that are phylogenetically and/or physiologically constrained, have been linked to corresponding process rates in nature (Schimel and Schaeffer, 2012). Schimel and Gulledge (1998) proposed studying methane-cycling microbial communities to demonstrate the connection between microbial community composition and ecosystem function. Environmental and climatic shifts can alter methane (CH_4_) flux profiles of soils (Bender and Conrad, 1992; Willison et al., 1995; Aronson and Helliker, 2010), likely through shifts in microbial community structure and function. Since the publication of Schimel and Gulledge (1998), numerous technological advances have allowed for the direct analysis of the connection between environmental and climatic factors and microbial community composition. In addition, our understanding of how different members of the microbial community contribute to soil CH_4_ flux has increased. In this review, we outline the responses of methane-cycling microbial community composition and abundance to environment and climate and how well these shifts correspond to changes in soil CH_4_ flux profiles.

The goal of this review is to highlight the current state of, and recent advances in, our understanding of CH_4_ consumption by microorganisms in terrestrial environments, as well as to point out areas where further study is needed. We hypothesized that net CH_4_ flux is correlated with the abundance and/or composition of methane-cycling microbes. We focus on non-wetland soils while touching on wetland and methanogen communities when relevant. To this end we discuss the main global changes that could impact methanotroph communities in particular. These changing environmental and climatic drivers include increased atmospheric CO_2_ and CH_4_ mixing ratios, increased temperature, changes in precipitation regimes, soil pH, and increased inorganic nitrogen (N) deposition to soil. In addition, we analyzed trends in CH_4_ fluxes by ecosystem, climatic zone, and vegetation type. In order to organize the body of knowledge on this topic, a meta-dataset was created from the literature, which is published along with this review as supplemental data. We believe that this dataset can assist in identifying future experimental directions as well as modeling efforts of the relationships between environmental and climatic changes, methane-cycling microbial communities, and soil CH_4_ fluxes.

### Background to the methane cycle

Methane is the 2nd most important anthropogenic greenhouse gas, responsible for 20–30% of total greenhouse gas radiative forcing since the industrial revolution (IPCC, 2007). Methane is currently about 200 times less concentrated in the atmosphere than is carbon dioxide, but each molecule of CH_4_ is 25 times more potent in terms of heat-holding capacity (Lelieveld et al., 1998). Due to changes in human activity and land use, both carbon dioxide and CH_4_ began to increase around 150 years ago, as the industrial age began. Since that time, atmospheric CH_4_ concentrations have increased ~150%; from a pre-industrial mixing ratio of about 0.7 ppm to ~1.8 ppm currently (Maxfield et al., 2006; Degelmann et al., 2010).

#### Variability in atmospheric methane concentrations

Atmospheric CH_4_ concentrations became erratic and did not increase overall from 1997 until 2007, and then began increasing again around 2008 (Rigby et al., 2008) and continue to increase. The reason(s) for this shift is unknown, but several explanations have been proposed for the recent vagaries in atmospheric CH_4_. Decreases in wetland sources have been proposed to explain the lack of growth in late 1990s and early 2000s (Bousquet et al., 2006). The patching of natural gas pipelines in Russia has also been proposed as an explanation for the change in atmospheric CH_4_ concentrations, since these had become leaky after the collapse of the Soviet Union, losing an estimated 29–50 Tg CH_4_ yr^−1^ in the late 1980s–early 1990s (Reshetnikov et al., 2000), although these numbers have not been confirmed. A reduction in fossil fuel sources has also been implied as the cause by a study of ethane levels in Greenland and Antarctic firn (Aydin et al., 2011). Also proposed are variations in atmospheric concentration of OH^−^ radicals (Rigby et al., 2008), yet there did not appear to be any increase in atmospheric CH_4_ destruction from these radicals recorded early in the duration of this decrease (Prinn, 2001) and there is an active debate over the reliability of past OH^−^ measurements (Lelieveld et al., 2004). Other explanations have focused on reduced rice agriculture and other microbial emissions, confirmed by isotopic measurements and models (Kai et al., 2011).

The wide range of potential explanations for past trends in atmospheric CH_4_ indicates a lack of understanding of the interplay between biotic and abiotic controls on CH_4_ cycling. The underlying biology of the microbial responses to environmental variables is still poorly understood (do Carmo et al., 2006). The non-wetland, terrestrial ecosystem CH_4_ sink may be larger than suggested by top-down models suggest, possibly accounting for this missing sink, but this hypothesis can only be tested with further study of soil methanotroph community composition and response to climatic and other variables. Indeed, the same isotopic fractionation evidence suggesting that reduced microbial sources may be responsible for the decline in atmospheric CH_4_ growth (i.e., Kai et al., 2011) could also imply increased microbial consumption. Small advances in our understanding of any CH_4_ source or sink will greatly improve our ability to budget this important greenhouse gas.

#### Atmospheric methane sources and sinks

**Figure 1:**
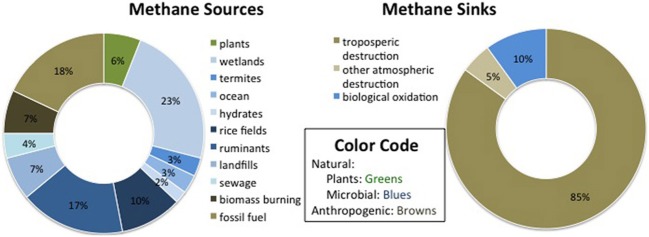
**Estimates of the relative contribution of sources and sinks to the global, annual methane budget**.

Methane sources are variable but their number and magnitude appear to be on the rise, while CH_4_ sinks are more uncertain. Total CH_4_ emissions were calculated by Lelieveld et al. (1998) to be 600 Tg CH_4_ yr^−1^, and by Wang et al. (2004) to be 506 Tg CH_4_ yr^−1^, with most recent estimates falling between 503 and 610 Tg CH_4_ yr^−1^ (IPCC, 2007). Figure 1 shows rough estimates of the relative contributions of CH_4_ sources and sinks, based on Lelieveld et al. (1998), Wang et al. (2004), and Conrad (2009). The largest global CH_4_ sources are natural and constructed wetlands, which contribute around 1/3 of annual emissions (IPCC, 2007). Anthropogenic sources, including rice paddies, domesticated animals, landfills, fossil fuel acquisition and burning, as well as biomass use for energy and agriculture, total at least 307 Tg CH_4_ yr^−1^, which could be over 60% of total emissions (Wang et al., 2004). There may be more sources than have been accounted for, as CH_4_ has also been found to be produced aerobically in the ocean (Karl et al., 2008). Trees themselves have also been linked to CH_4_ production (Keppler et al., 2006) through spontaneous UV-induced release and/or diffusion from dissolved soil CH_4_ in leaf water (Nisbet et al., 2009), although the overall contribution of that source has been shown to be negligible (Dueck et al., 2007).

There are indications that CH_4_ release from known sources was previously underestimated and has been on the rise with temperature increases in the last century. As high latitudes heat up in a generally warming climate, permafrost and accumulated ice thaw at accelerated rates (IPCC, 2007). This has caused the area of thermokarst lakes to increase, by at least double in the last 35 years (Walter et al., 2006). Advances in measurements in high latitude lakes show that most CH_4_ is released in rapid ebullition, a source type which was previously missed, and that the CH_4_ being released is Pleistocene in age, indicating the release of old carbon stores. This source accounts for at least 3.7 Tg CH_4_ yr^−1^ previously omitted from global estimates (Walter et al., 2006). Also associated with the warming in these higher latitudes is geological CH_4_ release from shallow hydrates, which may increase quickly as warming continues and could contribute up to 1.4 × 10^6^ Tg CH_4_ (Shakhova et al., 2010). Further, increased temperatures in wetlands around the globe will likely lead to large increases in CH_4_ release, due to the sensitivity of methanogens to warming (Christensen et al., 2003).

The largest estimated CH_4_ sinks include tropospheric destruction (approximately 80–90% annually) and oxidation in other parts of the atmosphere (5–10%), according to Lelieveld et al. (1998). The most common figure for gross oxidation by soil in terrestrial environments is ~30 ± 15 Tg CH_4_ (IPCC, 2007), which corresponds to 2.5–7.5% of the estimated 600 Tg CH_4_ budget per year (Lelieveld et al., 1998). However, there has been some variation in this estimate, with a classic review of methanotrophy estimating soil consumption at 40–60 Tg yr^−1^ (Hanson and Hanson, 1996). Of all the CH_4_ sources and sinks, the biotic sink strength is the most responsive to variation in human activities (Dunfield et al., 2007).

The above figures for total consumption by the soil were not measured directly, but rather approximated by top-down, or inverse, global models (Wang et al., 2004). Inverse modeling solves for the sources and sinks based on observations of atmospheric chemical species over time and space while attempting to minimize uncertainty (Prinn, 2000). More recently, a meta-analysis by Dutaur and Verchot (2007) attempted to scale up from averages of local observations, resulting in an estimated consumption rate of ~34 Tg CH_4_ yr^−1^. Due to low consumption levels at atmospheric concentrations and high variability, the bottom-up approach of extrapolating from small-scale observations has had limited success in the past. However, the bottom-up approach should be applied more strenuously in the near future to take advantage of advances in technology and more widespread measurements. Future attempts to scale up from local observations should also account for the environmental factors and their impacts on microbial communities that govern CH_4_ flux.

### Methane-cycling microorganisms

Soil exchange of CH_4_ with the atmosphere is regulated by two groups of microorganisms, known as methanogens and methanotrophs. The disparate environmental requirements of these two groups, particularly oxygen concentration, temperature, water content, and nutrient availability, determine the net CH_4_ flux of a given ecosystem. Methanogenic (CH_4_ producing) archaea, active mainly in anaerobic conditions, produce CH_4_ as a metabolic byproduct and are the main biological source of CH_4_ in natural systems, landfills, and agriculture. Methanotrophic (CH_4_ consuming) bacteria (sometimes referred to as CH_4_ oxidizing bacteria or MOB) are active mainly in aerobic conditions and derive energy and carbon from the oxidation of CH_4_ (Hanson and Hanson, 1996).

#### Methanogens

In natural systems, methanogens produce about 33% of emissions (Lelieveld et al., 1998). Most anthropogenic CH_4_ emissions from waste management and agriculture are also due in large part to the action of methanogens. Most methanogens are anaerobic archaea, and there exists a large variety of methanogens that loosely fit into two main, non-phylogenetic categories: those that are hydrogenotrophic, i.e., produce CH_4_ primarily using H_2_ and CO_2_; and those that are acetotrophic, i.e., use primarily acetate for metabolism that has been formed from previous decomposition activities (Le Mer and Roger, 2001). Most, if not all, known methanogens express an isozyme of methyl-coenzyme M reductase (MRT), of which the gene encoding the α subunit (*mcrA*) is present in most known methanogens (Shively et al., 2001).

#### Methanotrophs

The most common group of methane consumers is aerobic Methanotrophs (mostly methane oxidizing bacteria or MOB), which are generally found in oxic soils or microsites within anoxic soils. MOB are the only known biological sink for CH_4_, as key organisms within a soil microbial consortium that derives energy from CH_4_ conversion to carbon dioxide (Hanson and Hanson, 1996). Methanotrophs are a sub-group of the methylotrophs, which also contain methanol oxidizing bacteria (Kolb, 2009). There are 12 recognized genera of methanotrophs that are phylogenetically divided into type I (within the class *Gamma proteobacteria*) and type II (within the class *Alpha proteobacteria*; Mohanty et al., 2006). The key methanotrophic enzyme is CH_4_ monooxygenase (MMO), which occurs as both particulate (pMMO) and soluble (sMMO) forms. The *pmoA* gene encodes the α subunit of pMMO, and is included in the genome of all most known methanotrophic species (Dedysh et al., 2000). Methanotrophs are divided into at least two functionally distinct groups, the high affinity group that uses CH_4_ at very low concentrations, and the low affinity group that only uses CH_4_ at high concentrations (Bender and Conrad, 1992). Most culturable methanotrophs are low affinity, which tend to be located near source environments (Reay et al., 2005). In addition to the more common CH_4_ cyclers, a group of methanogen-like anaerobic CH_4_ oxidizing archaea (MOA) has been described (Hallam et al., 2003). These MOA contain *mcrA* genes (Hallam et al., 2003) and many are involved in a consortium that couples denitrification with anaerobic CH_4_ oxidation (Raghoebarsing et al., 2006).

## Microbial community composition impacts on methane flux

The capacity to produce or consume CH_4_ is distributed among relatively few microbial taxa that are phylogenetically distinct (Martiny et al., 2013). The narrow distributions of these traits imply that CH_4_ production and consumption rates may be more closely tied to microbial community composition and abundance than other biogeochemical processes (Schimel, 1995). Genes involved in methane-cycling are found in deep-branching microbial clades, similar to other complex microbial traits such as oxygenic photosynthesis and sulfate reduction (Martiny et al., 2013). By contrast, genes involved in heterotrophic processing of other carbon compounds are not highly conserved, and nearly all microbial taxa contribute to CO_2_ production in upland soils.

For methanogenesis, studies have found variation in the strength of the link between community structure and function. In a peat soil microcosms, methogenesis correlated positively with *mcrA* gene expression, which was a better predictor than gene abundance (Freitag and Prosser, 2009). The pathway of methane production shows a clear dependence on microbial composition, with acetoclastic methanogenesis dependent on the *Methanosarcinaceae* and CO_2_ reduction driven by groups such as the *Methanobacteriales* and *Methanosaetaceae*. These groups are sensitive to temperature, such that the CO_2_/H_2_ pathway becomes more dominant at higher temperatures (Fey and Conrad, 2000; Conrad et al., 2009). However, the temperature threshold for dominance varies from 15°C to 40°C across these studies, and both pathways are observed in peat soils with cooler average temperatures (Kotsyurbenko et al., 2004).

Other studies point to a more complex relationship between methane production and methanogen communities. Ramakrishnan et al. (2001) examined biogeographic patterns in methanogen communities across 11 rice field soils and found relatively similar microbial composition despite >10-fold differences in methane production rates. Similarly, Juottonen et al. (2008) observed relatively little change in methanogen abundance and composition across seasons in a boreal mire, but large variations in methane production that were likely due to increased substrate availability during winter. In a Siberian permafrost soil, Ganzert et al. (2007) found a shift from mesophilic to psychrophilic methanogens with depth, but no single group was clearly related to rates of methanogenesis, suggesting a degree of functional redundancy within methanogen communities.

As with methanogen communities, the link to functional rates is also variable for methanotroph communities. Some studies have found tight relationships between methane oxidation rates and community structure, often in the context of environmental change. In a temperate agricultural soil, long-term fertilization with ammonium nitrate reduced methanotroph abundance by >70%, resulting a similar decline in methane oxidation rates (Maxfield et al., 2008; Seghers et al., 2003a) observed a similar pattern that was associated with reductions in the abundance of low-affinity type I methanotrophs. Different groups of methanotrophs may show different responses to fertilization, as observed in rice field and forest soils where type II methanotrophs were more strongly inhibited by mineral N fertilization than type I methanotrophs (Mohanty et al., 2006). In contrast, organic fertilizer addition can increase methanotroph abundance and associated rates of methane oxidation (Seghers et al., 2005).

Gradient studies also suggest that variation in methanotroph abundance can correlate with functional rates. In a pine forest soil, methane oxidation rates across soil horizons were related to the abundance of a single PLFA marker identified with ^13^C stable isotope probing (Bengtson et al., 2009). Using a combination of molecular approaches and ^13^C tracers, Bodelier et al. (2013) found a tight link between methane consumption rates and the abundance of type 1 methanotrophs across a riparian floodplain. In contrast, studies in New Zealand have shown that type II methanotrophs are linked to higher methane oxidation rates associated with afforestation and reforestation (Singh et al., 2007; Nazaries et al., 2011). A similar pattern was observed across a broader gradient of vegetation types in Scotland, with increased type II methanotroph abundance, lower overall methanotroph diversity, and increased rates of methane consumption associated with forest vegetation (Nazaries et al., 2013).

Not all studies show such tight relationships between methanotroph communities and methane oxidation. Bárcena et al. (2011) found *pmoA* genes associated with high-affinity methanotrophs in a glacial forefield in Greenland, but detected almost no methane oxidation. Jaatinen et al. (2004) measured increased methane oxidation following boreal forest fire but no associated change in communities of methane-oxidizing bacteria. Conversely, Seghers et al. (2003b) found differences in methanotroph community composition but no substantial difference in methane oxidation in response to chronic herbicide treatment.

Differences in community composition that are not associated with differences in methane-cycling could indicate a degree of functional redundancy among methane-cycling microbes. However, such conclusions could be misleading. In some studies, more direct links between composition and function might have been observed if methanogen or methanotroph abundance had been measured. Studies using group-specific primers can identify within-group shifts in composition but not overall changes in abundance that may be more important for functional rates (Seghers et al., 2003a). For example, Menyailo et al. (2008) found that reductions in methanotroph-derived PLFA markers largely explained a 3-fold reduction in soil methane consumption following reforestation of a Siberian grassland. Despite the overall reduction in biomass, there were no apparent shifts in methanotroph community composition.

In addition, microbes that appear functionally redundant in one environment may show distinct responses when the environment changes. For example, different methanotroph communities may oxidize CH_4_ at similar rates in unfertilized soils (Seghers et al., 2003a), but communities dominated by type II methanogens could show much steeper declines in CH_4_ oxidation in response to N deposition (Mohanty et al., 2006).

Overall, many studies we reviewed support the idea that CH_4_ cycling depends on the composition and abundance of relatively narrow microbial groups. In addition, these studies demonstrate that environmental factors are important because they influence microbial communities. The abundances of methane-cycling microbes are often sensitive to environmental conditions such as temperature, precipitation, nutrient availability, CH_4_ concentration, and plant species (Fey and Conrad, 2000; Henckel et al., 2000; Horz et al., 2005; Liebner and Wagner, 2007; Maxfield et al., 2008; Tsutsumi et al., 2009). In some cases, these factors impact CH_4_ cycling though changes in microbial communities, but in other cases, environmental changes have important direct effects. For example, substrate availability and temperature both affect CH_4_ cycling rates, independent of changes in community composition (Wagner et al., 2005; Juottonen et al., 2008). Thus, even if CH_4_ cycling depends on narrow groups of methanogens and methanotrophs, the relationship between structure and function will always be subject to modification by environmental factors (Nazaries et al., 2011). This complexity will require models of the CH_4_ cycle that allow for feedbacks between microbial communities and environmental drivers.

## Environmental factors and the methane cycle

There is no ecosystem for which all of the potential direct or indirect effects of environmental variables on CH_4_ consumption of soil are understood, but many known interactions are summarized in Table 1. Conspicuously absent in Table 1 are any trends in tropical grasslands or savannahs, as there were no studies available testing environmental effects in these ecosystems to review. In general, the effect of higher soil moisture and precipitation is a decrease in the sink strength of the soil, however as Table 1 shows, even these impacts are not completely consistent. Other environmental variables that indirectly affect CH_4_ flux due to their influence on soil moisture and oxygen content are aspect and catena position, position on slope, soil type, and water holding capacity. Due to varying microbial preferences in terms of optimal pH, there is also some variation in response of CH_4_ flux to varying pH in the soil. Few general studies of distribution and activity of soil microbes as a whole have been done across catenas, slopes, or soil types, and many of those that have been done have not included methanotrophic or methanogenic organisms (Florinsky et al., 2004).

**Table 1:** **Summary of the impact of major environmental characteristics on methane uptake by soil**.

**Ecosystem/Biome**	**H_2_O Content**	**Precipitation**	**Position on slope**	**pH**
Boreal forest	low > high^1^ high > low^2^	low > high^3^	high > low^4^ low > high^5^	ND^6^
Boreal Steppe/Tundra	NR	low > high^7^	low > high^8^	high > low^9^
Temperate forest	low > high^10^ ND^11^	low > high^12^ ND^13^	high > low^14^ low > high^15^	high > low^16^ low > high^17^
Temperate grassland	low > high^18^	low > high^19^	high > low^20^ ND^21^	NR
Tropical forest	low > high^22^	low > high^23^ high > low^24^	high/flat > low^25^ low > high^26^	high > low^27^
Shrubland/Desert	high > low^28^	low > high^29^ ND^30^	NR	high > low^31^

*High and low refer to the variables in the column headers*.

*does not include agricultural systems except tree plantations; NR indicates that there were no studies located reporting on the indicated effect in that ecosystem/biome; ND indicates those studies that found no difference in CH_4_ flux with different values for that variable*.

1 Adamsen and King, 1993; Borken and Beese, 2006,

2 Ambus and Christensen, 1995; van Huissteden et al., 2008,

3 Bowling et al., 2009; Koide et al., 2010,

4 Borken et al., 2003,

5 Sjogersten and Wookey, 2002; Borken et al., 2003,

6 McNamara et al., 2008,

7 West et al., 1999; Mariko et al., 2007,

8 Sjogersten and Wookey, 2002,

9 Menyailo et al., 2008,

10 Castro et al., 1994, 1995; Klemedtsson and Klemedtsson, 1997; Prieme et al., 1997; Butterbach-Bahl and Papen, 2002; McLain et al., 2002; Borken et al., 2006; Rosenkranz et al., 2006; Aronson et al., 2012,

11 Prieme et al., 1997; Groffman et al., 2006,

12 Castro et al., 1994; Bradford et al., 2000; Blankinship et al., 2010a; Xu and Luo, 2012,

13 Borken et al., 2006,

14 Castro et al., 1993; Hart, 2006,

15 Yavitt et al., 1990,

16 Born et al., 1990; Brumme and Borken, 1999,

17 Sitaula et al., 1995; Prieme et al., 1997; Kolb et al., 2005,

18 Neff et al., 1994; van den Pol-van Dasselaar et al., 1998,

19 Blankinship et al., 2010b,

20 Mosier et al., 1991; Torn and Harte, 1996; Mosier et al., 1997a,b,

21 Brady and Weil, 1999; Chen et al., 2011

22 Keller et al., 1990; Jauhiainen et al., 2005; Teh et al., 2005; Konda et al., 2010,

23 Werner et al., 2006,

24 Davidson et al., 2004,

25 Delmas et al., 1992; Singh et al., 1997; Verchot et al., 2000; Wolf et al., 2012,

26 Silver et al., 1999,

27 King and Nanba, 2008,

28 Angel and Conrad, 2009,

29 Anderson and Poth, 1998; Galbally et al., 2010; Hou et al., 2012,

30 Blankinship et al., 2010a,

31 *Angel and Conrad, 2009*.

### Methane flux responses to increased methane concentrations

Although the average mixing ratio of CH_4_ at the Earth's surface has risen from around 0.7 ppm during pre-industrial times to about 1.8 currently, there has been little direct study of the impacts of rising atmospheric CH_4_ on the rate of consumption of CH_4_ by upland soils. Bender and Conrad (1992) determined that there were two kinetic optima for methanotrophy. There was a clear increase in the consumption of CH_4_ by the soil with increasing CH_4_ concentrations, indicating that the reaction is methane-limited at atmospheric oxygen levels (Bender and Conrad, 1992). However, they did not test consumption at CH_4_ concentrations between 2 and 6 ppm, since this range is thought to fall between the two V_max_ values for methanotrophy. Yet, this range might be relevant for soil CH_4_ consumption rates under global change. Most other investigations of methanotrophy responses to CH_4_ concentration have used high concentrations, focused either on determining kinetic or potential rates of methanotrophy (Henckel et al., 2000; Tuomivirta et al., 2009; Tate et al., 2012).

Recently, one study showed that levels of CH_4_ only slightly elevated above ambient can lead to markedly increased CH_4_ consumption. Irvine et al. (2012) observed a strong direct relationship between ambient CH_4_ concentrations at the start of CH_4_ flux measurement and the rate of consumption in salt marsh soils. This result could indicate that increases in average ambient CH_4_ concentrations will lead to a measurable increase in atmospheric CH_4_ consumption across soils.

### Methane flux responses to increased CO_2_ concentrations

Increases in CO_2_ can lead to increased methanogeny, both indirectly through greater biomass production increasing acetotrophic metabolism, and directly from CO_2_ stimulating hydrogenotrophic metabolism. In wetland areas the increased plant production due to elevated CO_2_ leads to greater CH_4_ release, likely due to acetotrophic metabolism (Dacey et al., 1994). Experiments in rice system soils have overwhelmingly agreed with these results (Ziska et al., 1998; Groot et al., 2003; Cheng et al., 2006). Whole soil and plant-facilitated emission of CH_4_ increased up to 69% in a wetland glasshouse experiment with elevated CO_2_ (Vann and Megonigal, 2003). However, plant facilitation may not add to this increase at all, as emissions facilitated by transport through wetland plants were not found to be changed by increased CO_2_ in a free-air CO_2_ enrichment (FACE) experiment (Baggs and Blum, 2004).

Though not as widely studied in non-wetland ecosystems, a similar trend was observed in two FACE studies performed in temperate forests, where heightened CO_2_ exposure resulted in an overall annual decrease in CH_4_ uptake of up to 30% (Phillips et al., 2001) and 25% (McLain et al., 2002). Another FACE study in a temperate grassland also showed decreased consumption with elevated CO_2_ (Ineson et al., 1998). It was hypothesized that these shifts were due to stimulation of methanogenesis by increased soil moisture in the lower soil layers (McLain et al., 2002; McLain and Ahmann, 2008; Dubbs and Whalen, 2010). However, elevated CO_2_ caused decreased overall bacterial counts and *pmoA* abundances (by qPCR and FISH) in a meadow soil (Kolb et al., 2005), indicating direct negative impacts on methanotrophy. Some studies have contradicted this trend, such as an open top chamber experiment in a shortgrass steppe, which showed a slight increase in net CH_4_ uptake that was not significant (Mosier et al., 2002). Similarly, elevated CO_2_ increased CH_4_ consumption in a grassland greenhouse study (Dijkstra et al., 2010). More analysis of the impact of elevated CO_2_ on CH_4_ flux in non-wetland terrestrial systems is needed before definitive conclusions can be drawn, specifically in the presence of other predicted global changes, such as warming.

### Soil moisture

Studies of precipitation and soil moisture content show correlations between wetter sites and decreased CH_4_ uptake or increased release (see Table 1), which is due in large part to the disparate environmental requirements of methanotrophs and methanogens. Throughfall exclusion in the Amazon basin caused CH_4_ consumption to more than quadruple compared to plots receiving natural precipitation levels (Davidson et al., 2004). Many studies have found that increased soil moisture content negatively influences CH_4_ consumption in ecosystems ranging from boreal, temperate, and tropical forests to shortgrass steppe, temperate farmland, and tundra (Adamsen and King, 1993; Castro et al., 1994; Klemedtsson and Klemedtsson, 1997; Epstein et al., 1998; Burke et al., 1999; West et al., 1999; McLain et al., 2002; Mosier et al., 2002).

However, there are intricacies that this generalization does not address. A dry tropical forest study showed that in the rainy season, CH_4_ consumption was inversely related to water content and precipitation (Singh et al., 1997). In the dry season, the trend was reversed, likely because all microbial activities are decreased, and the input of rain to severely dry soil leads to an increase in microbial activity, including methanotrophy. Boreal forest sites without peat show no significant difference in CH_4_ fluxes between inundated and dry soils. However, inundated peat soils released significantly more CH_4_ than dry peat soils from the boreal forest (Oelbermann and Schiff, 2010), indicating a vital role of water holding capacity of soil and surrounding vegetation.

#### Position in landscape, aspect, and catena

Factors such as position in the landscape, aspect, and catena impact CH_4_ flux indirectly, due to their impact on soil moisture retention. A mixed shrub, herb, and tree community showed higher CH_4_ consumption on North facing slopes (Burke et al., 1999). In a tundra study the results were mixed, with low snowmelt areas with high wind showing higher CH_4_ consumption on the North facing slope and areas with more snowmelt and protection having lower consumption on North facing slopes (West et al., 1999). A study in the boreal forest, using many different measures of CH_4_ flux and different tree communities showed that CH_4_ consumption was consistently greater on South facing slopes (Whalen et al., 1992). South facing slopes may have higher rates of evaporation than North facing slopes in the Northern Hemisphere, where all of these studies were located. This difference should lead to higher CH_4_ consumption on South facing slopes for more saturated soils, with the opposite effect for low water content soils, which does explain the mixed results seen in West et al. (1999). However, other factors may impact the effect of slope aspect, such as whether one slope receives higher precipitation due to orographic effects, as is known to occur in the Rocky Mountains of North America.

The impact of slope position is more variable, and more complete information is summarized in Table 1. For example, in the rainy season, dry tropical forest showed decreased CH_4_ uptake with low position on slope, with no trend in the dry season (Singh et al., 1997), which was also seen in boreal forest stands (Gulledge and Schimel, 2000). This result is likely due to prolonged increases in soil water content corresponding to poor drainage conditions and lower exposure to evaporation at low slope positions relative to hilltops. In Puerto Rican rainforest, the higher cloud forests release copious amounts of CH_4_, compared to the lower Tabanuco and Colorado forests which consume and release small amounts of CH_4_, respectively (Silver et al., 1999).

#### Soil type

Soil type exerts strong controls on the water holding capacity of soil, as well as the diffusion of gases into soil, both of which lead to pronounced effects on CH_4_ flux. Sandy soil (soil with larger particle size) has the lowest water holding capacity, followed by loam and then clay (Brady and Weil, 1999). The sand content of temperate grassland has been correlated with CH_4_ consumption rates, with sandy soil consuming more CH_4_ than loam, which in turn consumed more than clay (Born et al., 1990). Across terrestrial ecosystems, a recent meta-analysis performed by Dutaur and Verchot (2007) found that soil texture was one of the main factors correlated with CH_4_ fluxes, with coarser and medium-textured (loam) soils consuming more CH_4_ than fine (clay) soils (Dutaur and Verchot, 2007). Due to this recent meta-analysis, further discussion of the impact of soil type is limited in this review.

### Soil temperature

The methane-cycling microorganism response to temperature varies more than the response to changes in soil moisture. Insofar as temperature can lead to greater evapotranspiration, it may lead to decreased soil moisture, which would increase CH_4_ consumption. This trend was seen in multiple studies in temperate and boreal forests, which have found that higher observed soil temperatures correlate with greater uptake rates of CH_4_ (Castro et al., 1995; Klemedtsson and Klemedtsson, 1997; Bradford et al., 2001; Butterbach-Bahl and Papen, 2002; Rosenkranz et al., 2006). However, the enzymes involved in CH_4_ oxidation have variable optimum temperatures, with the average optimum temperature at 25°C (Hanson and Hanson, 1996). The enzymes involved in the degradation of organic matter that eventually results in methanogenesis have optima between 30 and 40°C (Le Mer and Roger, 2001). Similarly, temperature and precipitation have been shown to change the standing and ephemeral microbial community structure (Pritchard and Rogers, 2000), with varied consequences. A soil warming study using infrared heating, a method that provides a good approximation of future global warming (Aronson and McNulty, 2009), found that with increases in growing season temperature of up to 4.1°C there was no change in the CH_4_ flux of bog and fen mesocosms (Updegraff et al., 2001). However, higher temperatures (21°C vs. 14°C) caused significantly greater CH_4_ release from inundated peat soils from the boreal forest (Oelbermann and Schiff, 2010). Results were similar in a soil warming study within a grassland system, with increased heating causing lower CH_4_ uptake rates (Christensen et al., 1997).

### Nitrogen and fertilizer in the methane cycle

Global inorganic N input to non-wetland ecosystems from deposition, industry, and fertilizer use is projected to double from the 1990 levels by the year 2050 (Kroeze and Seitzinger, 1998). The effects of N on CH_4_ uptake in the soil environment are more complex than other environmental variables. Compared to natural forest and grassland, cropland and pasture consume less CH_4_ and show greater decreases in CH_4_ consumption rates with increased nitrogen additions (Aronson and Helliker, 2010). In general, the conversion of native lands to row-crop agriculture has been found to lead to a seven-fold reduction in both methanotroph diversity and CH_4_ consumption (Levine et al., 2011). The genetics and enzyme kinetics behind CH_4_ oxidation show tight evolutionary and functional linkages between the enzymes that enable CH_4_ and ammonia oxidation (Dunfield and Knowles, 1995). Methanotrophs and ammonia oxidizers are capable of switching substrates, which is a mechanism believed to be responsible for the inhibition of CH_4_ uptake by soil exposed to high concentrations of ammonia (Hanson and Hanson, 1996). In a rice paddy soil, CH_4_ oxidation and nitrification (i.e., ammonia oxidation) were inversely related in the presence of high N (Alam and Jia, 2012). In a wetland study by Baggs and Blum (2004), emissions facilitated by transport through plants were doubled with a four-fold increase in N deposition. However, laboratory experiments at elevated levels of ammonium showed that the inhibition of CH_4_ oxidation did not correspond to a shift in methanotroph communities (Bykova et al., 2007).

Methanotrophs demonstrate N limitation of CH_4_ uptake at low concentrations of available nitrogen relative to available CH_4_ in both N-limited wetlands (Bodelier et al., 2000) and upland soils (Aronson et al., 2012). A potential mechanism for this observed stimulation of CH_4_ oxidation with added inorganic N, in N-limited systems, was proposed by Bodelier and Laanbroek (2004) to be the N-fixation pathway found in a subset of methanotrophs, specifically the nitrogenase pathway found in types II and X methanotrophs (Hanson and Hanson, 1996). Type X methanotrophs are closely related to type I, but share some metabolic similarities with type II (Macalady et al., 2002). Thus, it has been put forward that in N-limited conditions, methanotrophy is limited by the energy requirement of N fixation (Henckel et al., 2000). Evidence for stimulation of methanotrophy by addition of low levels of inorganic N has been found in some non-wetland terrestrial systems (Aronson and Helliker, 2010). In general, soil drainage condition may indicate whether N stimulates methanotrophy, inhibits it, or does not impact the CH_4_ cycle at all (Aronson et al., 2013).

### Soil pH

Methanotrophs are more sensitive to acidic environments than are methanogens, although they are more tolerant of variations in pH through time (Le Mer and Roger, 2001). With the exception of variable responses to pH in the temperate forest, there was a general trend of increasing CH_4_ consumption with higher pH (Table 1). There was also no clear trend in the boreal forest studied (McNamara et al., 2008).

## Ecosystem and vegetation effects on methane uptake

**Figure 2:**
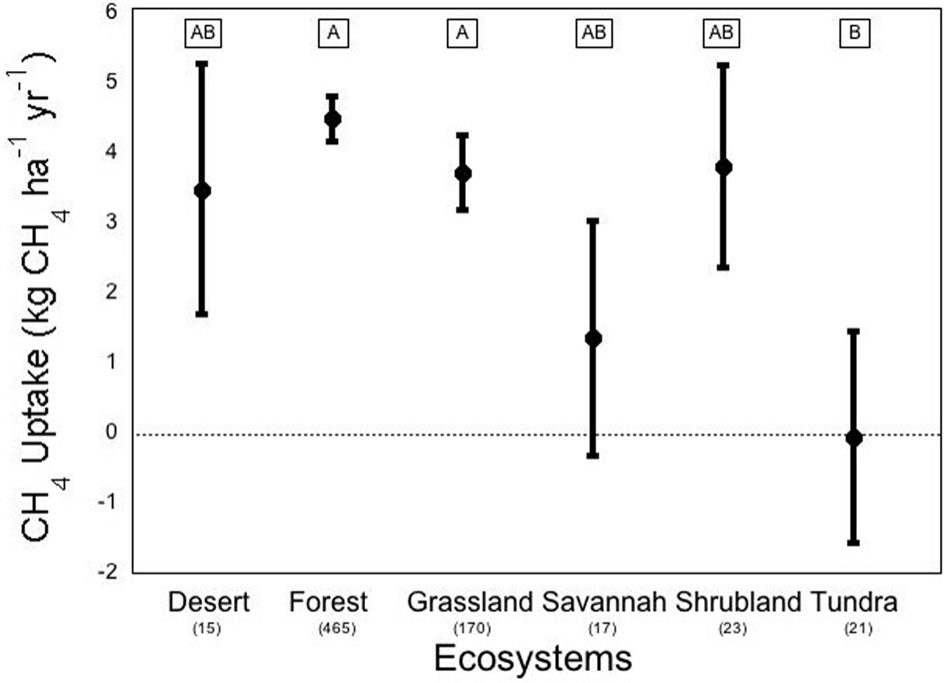
**Methane flux by ecosystem**. Negative numbers indicate net release of methane by the soil. Averages are expressed bounded by standard errors of the means. The number of studies included in each average is listed in parentheses under each ecosystem type. Means with the same letter are not significantly different (Student's *t*-test).

**Figure 3:**
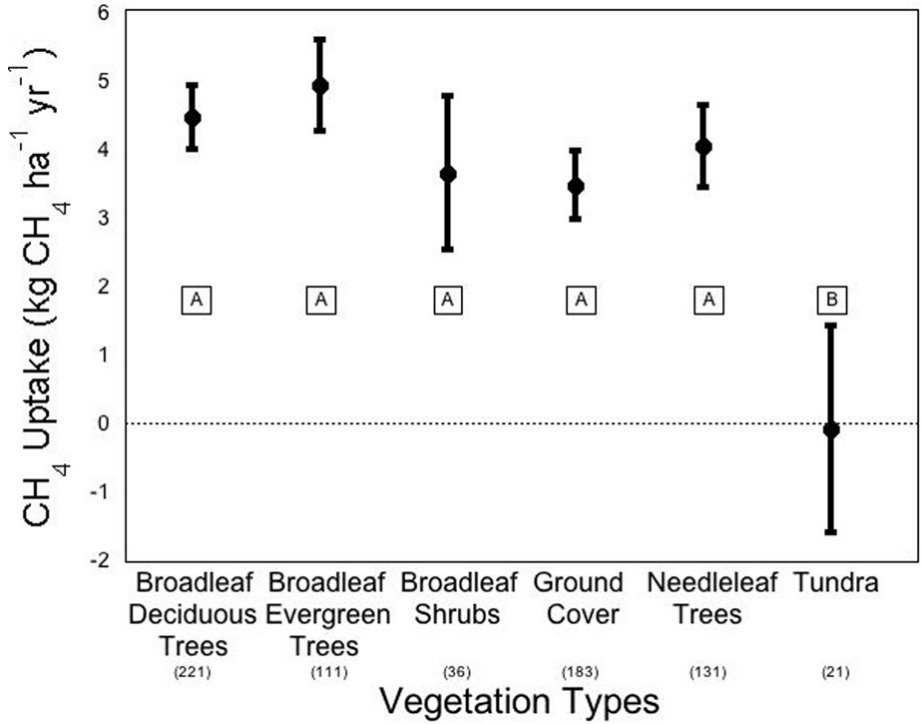
**Methane flux by vegetation types**. Negative numbers indicate net release of methane by the soil. Averages are expressed bounded by standard errors of the means. The number of studies included in each average is listed in parentheses under each vegetation type. Means with the same letter are not significantly different (Student's *t*-test).

We conducted a meta-analysis to determine ecosystem and vegetation impacts on CH_4_ uptake in upland soils (methods in Appendix A, database in Appendix B). Across the ecosystems included in our meta-analysis, there exists a high variability in CH_4_ flux by ecosystem type (Figure 2). The One-Way ANOVA performed across studies by ecosystem type found that there was a significant difference between ecosystem types (*p* < 0.031). Means comparisons using Student's *t* revealed that forests and grasslands consumed more CH_4_ than tundra, with the other ecosystems not different from each other. In addition, vegetation type (Figure 3), was significant by ANOVA (*p* < 0.044). Means comparisons showed that tundra, which released methane on average, differed significantly from all other vegetation types, which consumed methane.

On average, forest systems show the greatest CH_4_ consumption capability of any ecosystem, at an average of about −4.50 ± 0.32 kg ha^−1^ yr^−1^. The variation between forest observations is great, even though the standard error is relatively low, due to the fact that the number of studies included in the database from forests is an order of magnitude greater than most other ecosystems. This rate can be much higher; a study of a New Zealand pine forest found an overall uptake of CH_4_ at an annual rate of –12.1 kg ha^−1^ yr^−1^ (Tate et al., 2006). At the extreme end, an early CH_4_ uptake study in a British mixed-temperate forest on a single day found an uptake rate that would scale to –165 kg ha^−1^ yr^−1^ (Willison et al., 1995). But not all forests consume CH_4_ overall; a study of the CH_4_ budget of a black spruce forest in Germany found an average CH_4_ release of 54.5 kg ha^−1^ yr^−1^ (Fiedler et al., 2005). Tundra ecosystems (including “alpine” and “subarctic” tundra) on average were found to release CH_4_ at a rate of 0.035 kg ha^−1^ yr^−1^. Tundra also displayed extremely high variation in uptake rates across various environmental conditions, which may be due to ebullition; the release of large amounts of CH_4_ in bubbles from clathrate associations deep below the soil or water column (Shakhova et al., 2010). Vegetation height has also been found to be a good indicator of CH_4_ release in varied wet tundra sites (von Fischer et al., 2010). Deserts displayed the greatest variation, with mean ± standard error of desert flux found to be 3.49 ± 1.79 kg ha^−1^ yr^−1^ across 9 studies, which may be due to more extreme responses to precipitation pulses. Alternately, this variation may be due fact that deserts over natural gas deposits have been shown to be CH_4_ sources (Etiope and Klusman, 2010).

### Vegetation effects

Robust differences in CH_4_ fluxes appear when separated by vegetation type (Figure 3; ANOVA *p* = 0.009). Individual plant species effects on CH_4_ flux can be substantial, but most effects have been reported in wetland species. The most common species effects occur in some wetland plants that facilitate CH_4_ entering and leaving the soil or sediment. For an example with the sedge plant type/functional type, there is a clear difference between *Carex scopulorum*, which allows the emission of CH_4_, and *Kobresia myosuroides*, which allowed the consumption of CH_4_ (West et al., 1999). Confounding may frequently emerge in most experiments that report on the plant species and functional type causes of uptake because the effects of plant species are difficult to tease apart from the effects of environmental variables, which may in turn predict plant species colonization. For example, in West et al. (1999), the variation in amount of snowmelt received during the snow-free months in the alpine tundra predicted plant species dominance differences. The CH_4_ uptake rate in these sites varied, but whether the variation was due to a species or environmental effect is ambiguous (West et al., 1999).

Generally when plant effects are observed, it is not specific species but plant functional type differences that are of interest, with the soil around trees associated with higher CH_4_ consumption than shrubs, grasses, and sedges. Across studies, deciduous forests have higher CH_4_ uptake rates than do coniferous forests (Degelmann et al., 2010), which is likely related to pH impacts. In the meta-analysis, we found broadleaf deciduous trees to consume −4.51 kg CH_4_ ha^−1^ yr^−1^ compared to –4.08 kg CH_4_ ha^−1^ yr^−1^ in needleleaf trees, however, this difference was not significant (Figure 3). There was also one study that directly tested the impact of tree proximity on CH_4_ uptake rate and found that there is greater net uptake by soils that are closer to deciduous trees and further from coniferous trees (Butterbach-Bahl et al., 2002). There has also been an observed effect of grass functional diversity on CH_4_ uptake in shortgrass steppe soils (Epstein et al., 1998). In clay soils, a mixture of C_3_ and C_4_ grasses appeared to consume more CH_4_ than either grass type alone, though these results were not significant at the 5% level. In sandy clay soils, a different effect was observed with C_4_ plants significantly increasing uptake of CH_4_ compared to C_3_. Mixed grasses fell between the grass types and did not differ significantly from either C_3_ or C_4_ uptake (Epstein et al., 1998).

### Disturbance, burning, and plant succession

There has been limited study of the impacts of burning, grazing, plant removal, and other disturbances on CH_4_ uptake by soils. There are no clear trends in a handful of studies on the effects of burning on CH_4_ flux performed across multiple ecosystems. In tropical forests and temperate grasslands, burning increased consumption of CH_4_ (Tate and Striegl, 1993; Poth et al., 1995). Burning results in vegetative cover removal that could increase the sunlight reaching the soil, therefore allowing for a lower water filled pore space and more consumption of CH_4_. However, in tropical savannas the impact of burning was decreased consumption (Prieme and Christensen, 1999). In boreal forests and Mediterranean shrublands, the response to fire was mixed or there was no change at all (Gulledge et al., 1997; Anderson and Poth, 1998; Castaldi and Fierro, 2005).

The impact of non-fire vegetative removal has also been mixed across ecosystems. Grazing has been shown to increase CH_4_ uptake in the boreal steppe (Geng et al., 2010). In temperate and tropical grasslands grazing generally decreased consumption (Zhou et al., 2008; Chen et al., 2010, 2011; Wang et al., 2012). Clipping was found to increase CH_4_ consumption in tropical savannah (Sanhueza and Donoso, 2006). Thinning of the trees decreased CH_4_ consumption in one temperate forest (Dannenmann et al., 2007), but not another (Wu et al., 2011). Clear-cutting reduced consumption in the boreal forest (Saari et al., 2004) and temperate forest (Wu et al., 2011).

Changes in CH_4_ consumption are often observed during ecological succession following disturbance. Within forests, the climax (i.e., virgin or old-growth) vegetation is most often found to consume more CH_4_ than early successional stages This trend was found in two temperate forest studies of deciduous (Hudgens and Yavitt, 1997) and mixed deciduous and coniferous stands of various ages since disturbance (Brumme and Borken, 1999). Within tropical forests, old-growth forest was found to consume more CH_4_ (Keller and Reiners, 1994; Verchot et al., 2000; Veldkamp et al., 2008; Zhang et al., 2008). MacDonald et al. (1999) had mixed results and MacDonald et al. (1998) and Goreau and Mello (1985) found that secondary forest consumed more CH_4_ than old-growth forest. (Kruse and Iversen, 1995) found that in temperate grasslands, post-plow secondary growth soils consumed more CH_4_ than both bare plowed soil and natural heathland. They also found that oaks invading the grassland consumed resulted in more CH_4_ consumption than the nature heathland or secondary grasses, and that old-growth and established oak stands consumed even more CH_4_ (Kruse and Iversen, 1995). In Mediterranean shrublands, old growth shrubs consumed more CH_4_ than early and mid-succession (Price et al., 2010).

## Conclusions

Methane-cycling microorganisms in soils have the potential to impact the atmospheric composition of the Earth. As a narrow process, we found the composition of the microbial communities responsible for CH_4_ consumption and production have been linked to corresponding process rates in nature, as was proposed by Schimel and Gulledge (1998). We hypothesized that net CH_4_ flux would be correlated with the abundance and/or composition of methane-cycling microbes. In fact we found prolific, although not entirely consistent, evidence that the impacts of environmental and climate drivers on net CH_4_ flux are the result of changes in the methane-cycling microbial community. However, we found fewer studies that linked these changes to overall abundance of methanotrophs and/or methanogens, or specific phylogenetic lineages within these groups. This is an area of study ripe for investigation, and we believe that coupled with the knowledge of the impact of shifts in community composition, this data on abundance could complete the picture of the role of microorganisms in the global CH_4_ cycle.

Combined with information on microbial community impacts on CH_4_ flux, the dataset created for this review can assist in future modeling efforts. In particular, it demonstrates relationships between environmental and climatic changes, methane-cycling microbial communities, and soil CH_4_ fluxes. Process-based and ecosystem-specific models of CH_4_ flux are necessary to predict ecosystem CH_4_ fluxes in response to environmental and climatic changes. In order to create these models, certain ecosystems deserve further study, either because they consume large amounts of CH_4_ or because they are understudied. In particular, attention should be focused tropical grasslands and savannahs. Secondarily, some attention should be paid to the impact of pH in boreal forest and soil moisture content in boreal steppe/tundra, as well as the impacts of temperature across the boreal landscape, as research on these topics is lacking and most warming is expected to occur in high latitudes where these ecosystems are prevalent (IPCC, 2007).

Finally, it is important to decrease the uncertainty regarding CH_4_ sources and sinks in order to improve predictions of future global warming. We now have the tools necessary to answer questions about recent fluctuations in the CH_4_ growth rate in the atmosphere and predict the CH_4_ budget. The increasing use of eddy covariance techniques for regional scale estimates of CH_4_ fluxes can assist these global inventories, but should be paired with chamber-based flux measurements to account for the effects of environmental variation. Small-scale process-based models, global inventories, and global inverse models have all approached this issue with limited success. The next generation of models must use process-based and microbial community knowledge to account for seasonal and inter-annual variation in global CH_4_ budgets.

### Conflict of interest statement

The authors declare that the research was conducted in the absence of any commercial or financial relationships that could be construed as a potential conflict of interest.

## Appendix A

### Meta-analysis: methods of database creation

Methane flux data were extracted from published studies in non-wetland terrestrial ecosystems and farming systems, and are listed in Appendix B, using the same methods as in Aronson and Helliker (2010). Analysis was limited to the measurement of net flux due to combined methanogeny and aerobic oxidation of CH_4_ under ambient CH_4_ concentrations; uptake by anaerobic oxidation or under elevated [CH_4_] was not considered. All included studies used intact soil, mostly *in situ* with the exception of (Kruse and Iversen, 1995) and Willison et al. (1995), which used intact soil cores exposed to atmospheric CH_4_ concentrations soon after removal from the field. All studies used static (mostly vented) chambers (Hutchinson and Mosier, 2002) or flow-through auto-chambers (e.g., Brummell et al., 2012). All the original data were extracted from figures, tables, and text in the published papers. The studies were located using review papers (Le Mer and Roger, 2001; Dutaur and Verchot, 2007) and ISI Web of Knowledge using search terms: “methane” and “uptake,” “oxidation,” “flux” or “consumption.” In particular, all applicable studies from Dutaur and Verchot (2007) were included in the database if the original article could be located. Unpublished data from the dataset published with Dutaur and Verchot (2007) were not used.

The resultant database (Appendix B) from 194 papers, consisted of 716 entries, each containing a methane flux measurement matched with ancillary information. There were multiple entries from many studies due to differing environmental information associated with each methane flux measurement. The annual CH_4_ uptake averages presented in primary or secondary literature were used when applicable, while averages were calculated based on figures if no yearly average was provided. All flux measurements were standardized to a flux density of CH_4_ in kg ha^−1^ yr^−1^. Ancillary information from each data source, included: latitude, longitude, and location information; average annual temperature and precipitation; elevation; soil type or description; duration of study; start year; climatic zone; ecosystem type (as described in the reference); vegetation type; season(s) studied; environmental and fertilization information; replication information; plant type and species; and collection method and intervals.

The ecosystem types in Figure 2 were gathered from the references. The climatic zones were also taken from the references, when this information was provided. When the reference did not state climatic zone, it was based on latitude (up to 25 degrees was considered tropical, 25°–50° was considered temperate, and 50°–70° was boreal, greater than 70° was tundra). The vegetation types from Figure 3 are groupings of the dominant plants associated with each methane flux measurement. The types broadleaf deciduous, broadleaf evergreen, and needleleaf trees were taken directly from plant types the text, or inferred based on the ecosystem type listed. However, broadleaf shrubs were considered to include desert vegetation, chaparral and some grassland-type sites where shrubs were listed as dominant, in addition to shrubland. Tundra vegetation was variable, and the vegetation type classification was always given to that ecosystem/climatic zone. Ground cover included grasslands, heathland, steppe, and savannah. Agricultural systems were excluded from the comparisons shown in Figures 2, 3, since their flux profiles may not follow with natural gradients. In some cases the numbers associated with each ecosystem and vegetation type (in Figures 2, 3) differ from the number of those methane flux measurements due to the removal of a 5 outlier points (an order of magnitude greater consumption or release than the others) for statistical purposes.

### Data analysis

All statistical analysis was performed using JMP Pro 10 (SAS, Inc.). The statistical tests performed included One-Way ANOVA, as well as *post-hoc* Student's *t*-test comparisons. The significance cut-off was *p* < 0.05.
